# Community-based evaluation of Saudi FRAX with and without BMD in Riyadh, Saudi Arabia

**DOI:** 10.1007/s11657-025-01638-w

**Published:** 2025-12-05

**Authors:** Muath Alkhunizan, Nouf Almasoud, Majd Abdulmowla, Zoha Khalid, Norah Albedah, Mohammad Elshaker

**Affiliations:** 1 Department of Family Medicine and Polyclinics, King Faisal Specialist Hospital and Research Centre, MBC 62, PO Box 3354, 11211 Riyadh, Saudi Arabia; 2 College of Medicine, Alfaisal University, Riyadh, Saudi Arabia; 3 Department of Hematology, Guy’s and St Thomas’ Hospitals NHS Trust, London, UK

**Keywords:** Osteoporosis, Bone mineral density, FRAX, Risk assessment, Fractures, Bone, Saudi Arabia

## Abstract

***Summary*:**

Osteoporosis is a growing health burden requiring early risk assessment. In Saudi adults ≥ 60 years, FRAX scores with and without BMD showed minimal reclassification, with most shifts toward higher risk in younger groups. FRAX without BMD offers reliable stratification, supporting its use as a first-line screening tool.

**Methods:**

A retrospective cross-sectional study included patients aged ≥ 60 years attending family medicine clinics at King Faisal Specialist Hospital & Research Centre, Riyadh, undergoing DXA screening between January 1, 2016, and December 31, 2022. Baseline variables were age, sex, and BMI. FRAX variables assessed were family history of hip fracture (HF), prior fracture, smoking, glucocorticoid use, rheumatoid arthritis, and alcohol intake. Fracture risk was categorized using fixed and age-specific thresholds; differences in FRAX scores and reclassification patterns were evaluated.

**Results:**

Among 1,429 patients (mean age 68.07 ± 6.62 years), FRAX scores for major osteoporotic fracture (MOF) and HF) differed significantly with and without BMD. Subgroup analysis revealed no significant differences within specific age ranges (70–75 years for MOF and 60–75 years for HF) or among men across all age groups. Reclassification occurred in a small subset of patients, predominantly shifting toward higher-risk categories, particularly in younger age groups.

**Conclusion:**

FRAX without BMD provides robust risk stratification, with minimal impact on reclassification across age categories. These results highlight its utility as a primary screening strategy, whereas BMD assessment may be reserved for cases requiring further refinement.

**Purpose:**

Osteoporosis is a skeletal disorder defined by reduced bone mineral density (BMD), commonly assessed by dual-energy X-ray absorptiometry (DXA). The Fracture Risk Assessment Tool (FRAX) estimates 10-year fracture risk. Saudi Arabia was recently included in FRAX, enabling population-specific risk estimation. Notably, FRAX can be applied with or without BMD, but limited Saudi data exist comparing both methods.

## Introduction

Osteoporosis is a skeletal disorder characterized by reduced bone strength, leading to increased fragility and fracture risk [1]. A recent systematic review and meta-analysis reported a prevalence of osteoporosis in Saudi Arabia as high as 32.7%, significantly higher than the global prevalence of 18.3% [2, 3]. In comparison, the prevalence of osteoporosis across all age groups in the EU27 + 2 countries (the 27 European Union member states plus the United Kingdom and Switzerland) is estimated at 5.6% [4]. Recent Saudi study showed the prevenance of osteoporosis in older adults was 8.2% and 11.8% based on femoral and lumbar bone mineral density (BMD) measurements, respectively [5]. The disease imposes a substantial health and economic burden; the estimated annual direct cost per patient in Saudi Arabia is $975.77 without fracture, rising to $9,716.26 with fractures [6]. Fragility fractures, in particular, represent a major concern, as they are a leading cause of morbidity and mortality in osteoporotic patients [7]. In Saudi Arabia, the incidence of osteoporosis-related femoral fractures is estimated at 5.81 per 1,000 individuals over the age of 59 [8]. These findings highlight the urgent need for reliable fracture risk assessment tools to guide prevention strategies and reduce the morbidity and mortality associated with osteoporosis-related fractures.

The World Health Organization (WHO) defines osteoporosis as a bone mineral density (BMD) more than 2.5 standard deviations below the mean for a healthy young adult [9]. Although BMD is an important predictor of fracture risk [10], it alone cannot fully determine future fracture likelihood, as clinical factors such as age contribute independently to risk [11]. To address this, the FRAX tool was developed by the University of Sheffield under the supervision of the WHO. FRAX estimates the 10-year probability of fracture by integrating multiple clinical variables, including age, prior fractures, and glucocorticoid use. BMD, typically measured by dual-energy X-ray absorptiometry (DXA), can be incorporated into the model, although it remains optional [12]. Importantly, FRAX without BMD has been shown to be a valid alternative for fracture risk estimation [13].

The role of BMD in the FRAX tool remains controversial. Several studies have demonstrated that FRAX without BMD provides predictive accuracy comparable to FRAX with BMD [14–16]. In regions lacking DXA availability, FRAX without BMD has shown good sensitivity and acceptable specificity [17]. In contrast, other studies report that incorporating BMD improves fracture risk prediction compared to clinical risk factors alone [18]. The Saudi Osteoporosis Society (SOS) currently recommends using FRAX with clinical risk factors as a baseline, applying age-specific intervention thresholds, and reserving BMD assessment for patients at intermediate risk [19]. Considering the variability in findings across populations, external validation of FRAX with and without BMD in the Saudi context is essential.

The present study aims to compare FRAX scores calculated with and without BMD in Saudi Arabia and to determine which approach more accurately predicts future fracture risk, addressing the current gap in local evidence.

## Methodology

### Study design and setting

A retrospective cross-sectional study was conducted using data from patients aged ≥ 60 years who attended family medicine clinics and polyclinics at King Faisal Specialist Hospital & Research Centre (KFSH&RC), Riyadh, Saudi Arabia, between January 1, 2016, and December 31, 2022, and were screened for osteoporosis using DXA.

### Inclusion criteria and exclusion criteria

All patients aged ≥ 60 years who underwent DXA screening without a prior diagnosis of osteoporosis were included. Individuals with secondary causes of osteoporosis, such as liver disease, kidney disease, or malabsorption disorders, were excluded.

### Data collection and variables

Data were extracted from electronic medical records and organized into four domains: sociodemographic characteristics, FRAX-required clinical variables, BMD values, and FRAX scores. Data entered to Microsoft Excel (Microsoft Corp., Redmond, WA, USA). Sociodemographic variables included age, sex, height, weight, and BMI. Clinical factors required by FRAX comprised history of prior fracture, parental hip fracture (HF), smoking status, glucocorticoid use, rheumatoid arthritis, secondary osteoporosis, and alcohol intake (> 3 units/day). BMD was assessed by DXA measurement of t-scores at the lumbar spine and femoral neck. FRAX scores were then generated with and without incorporation of BMD.

### DXA measurement

BMD was assessed using GE Healthcare Lunar intelligent Dual-energy X-ray Absorptiometry (iDXA). Measurements were obtained at the lumbar spine (L1–L4) and bilateral hips (right and left proximal femur). The GE Lunar system provides individual and mean BMD values for both hips. T-scores were derived using the manufacturer’s Middle East female reference database. Quality control followed GE Lunar protocols, including daily phantom calibration, precision monitoring, and Least Significant Change (LSC) assessment at the 95% confidence interval.

### Details of FRAX tool requirements

Within the FRAX tool, certain clinical variables follow standardized definitions. A “previous fracture” refers to one that occurred spontaneously or from trauma insufficient to cause fracture in a healthy individual. “Glucocorticoid use” is defined as current or prior intake of ≥ 5 mg of prednisone (or equivalent) daily for more than three months. The tool accepts age inputs ranging from 40 to 90 years.

### Fracture risk classification and age group

For fracture risk classification, two sets of thresholds were applied:

- 1. **Age-specific thresholds** for major osteoporotic fractures (MOF) and HF were assigned according to the SOS’s 2023 clinical guidelines [19]. These thresholds were applied to FRAX-based risk estimates both without BMD and with BMD Risk categories were defined as:
  - Low risk: FRAX risk estimate below the lower age-specific threshold
  - Intermediate risk: FRAX risk estimate between the lower and upper age-specific thresholds
  - High risk: FRAX risk estimate above the upper age-specific threshold
- 2. **Fixed cutoffs from the literature** were also used for comparison, based on a previous study on fracture risk in women with early breast cancer [17]. In this classification:
  - HF risk was categorized as low risk (≤3%) or high risk (>3%)
  - Major osteoporotic fracture risk was categorized as low risk (<10%), moderate risk (10%–20%), and high risk (>20%)

These classification methods were applied separately for MOF and HF risk, both with and without BMD included in the FRAX calculation, to assess the impact of adding BMD on risk stratification.

### Statistical analysis

Statistical analyses were performed using Stata version 17 (StataCorp LLC, College Station, TX). Baseline characteristics of the study cohort (n = 1429) were summarized using descriptive statistics, with continuous variables expressed as means ± standard deviations (SD) and categorical variables as frequencies and percentages. Median FRAX scores for hip and MOF risks, with and without BMD, were compared using the Wilcoxon signed-rank test. Age-stratified analyses were also conducted with the same test, with age groups defined according to clinical relevance. A two-tailed p-value of < 0.05 was considered statistically significant.

## Results

Between 2016 and 2022, a total of 1,429 patients aged ≥ 60 years who attended the Family Medicine & Polyclinics Department at KFSH&RC, Riyadh, were retrospectively reviewed. Baseline characteristics are summarized in Table 1. The mean age of the cohort was 68.07 ± 6.62 years, with males representing 25.3% (n = 362). The average BMI was 30.90 ± 5.99 kg/m^2^. The mean lumbar spine BMD was 0.98 ± 0.17 g/cm^2^, and the mean femoral neck BMD was 0.80 ± 0.13 g/cm^2^. The mean T-score at the lumbar spine was − 0.92 ± 1.45, whereas the mean femoral neck T-score (right and left) was − 1.17 ± 1.01, reflecting variable degrees of bone loss across the study population.

**Table 1:** Descriptive statistics of bassline characteristics of the studied sample (n = 1429)

Characteristics	Total (n = 1429)(Mean ± SD)	Male (n = 362) (Mean ± SD)	Female (n = 1,067) (Mean ± SD)
Age (years)	68.07 ± 6.62	70.92 ± 6.98	67.10 ± 6.20
Height (cm)	157.42 ± 8.47	166.35 ± 7.13	154.40 ± 6.54
Weight (kg)	76.30 ± 14.34	77.48 ± 14.50	75.91 ± 14.28
BMI (kg/m^2^)	30.90 ± 5.99	27.95 ± 4.62	31.91 ± 6.08
BMD Lumbar Spine (g/cm^2^)*	0.98 ± 0.17	1.06 ± 0.21	0.97 ± 0.16
BMD Femoral Neck (g/cm^2^)	0.80 ± 0.13	0.83 ± 0.15	0.79 ± 0.13
T-Score Lumbar Spine*	− 0.92 ± 1.45	− 0.41 ± 1.70	− 1.10 ± 1.32
T-Score Femoral Neck	− 1.17 ± 1.01	− 1.33 ± 1.03	− 1.13 ± 1.00

*Missing 2 participants

Table 2 presents the median FRAX scores (with interquartile ranges) for hip and MOFs, calculated with and without BMD. Significant differences were observed between the two methods for both fracture types (*p* < 0.001). The median HF risk was marginally higher without BMD (0.6 vs. 0.5), whereas the median MOF risk was slightly higher with BMD (3.0 *vs.* 2.9). These results underscore the effect of incorporating BMD into FRAX-based risk estimation.

**Table 2:** Median FRAX scores for fracture cases with and without BMD

FRAX score of HF category	Median (IQR^1^)	*P*-value
FRAX score for *HF with* BMD	0.5 (0.2–1.0)	0.0005
FRAX score for *HF without* BMD	0.6 (0.4–1.0)	
FRAX score for *MOF with* BMD	3.0 (2.5–3.9)	0.0001
FRAX score for *MOF without* BMD	2.9 (2.3–3.6)	

*Wilcoxon Signed-Rank Test ^1^ 1 interquartile range

Table 3 shows the median FRAX scores for MOF and HF with and without BMD across age groups, with corresponding Wilcoxon signed-rank test p-values. Significant differences in MOF risk were observed at ages 60 (p < 0.0001), 65 (*p* = 0.0009), and 80 (*p* = 0.0107), whereas no differences were noted at ages 70 and 75. For HF risk, only the 80-year group demonstrated significance (*p* = 0.0002). These findings indicate that the influence of BMD on FRAX estimates varies by age, with greater impact at younger (MOF) and older (HF) ages.

**Table 3:** Comparison of median FRAX scores for fracture cases with and without BMD within different age groups

Age (years)	Median MOF Score			Median HF Score		
	With BMD	Without BMD	*P*	With BMD	Without BMD	*P*
≥ 60	3.00	2.80	*0.0000*	0.30	0.40	0.5790
65–69	3.30	3.20	*0.0009*	0.50	0.60	0.4303
70–74	3.30	3.10	0.6104	0.70	0.90	0.0615
75–79	2.95	3.10	0.9910	0.85	1.10	0.0570
> 80	2.2	2.35	0.0107	0.80	1.10	*0.0002*

*wilcoxon Signed-Rank Test

**Fig. 1:**
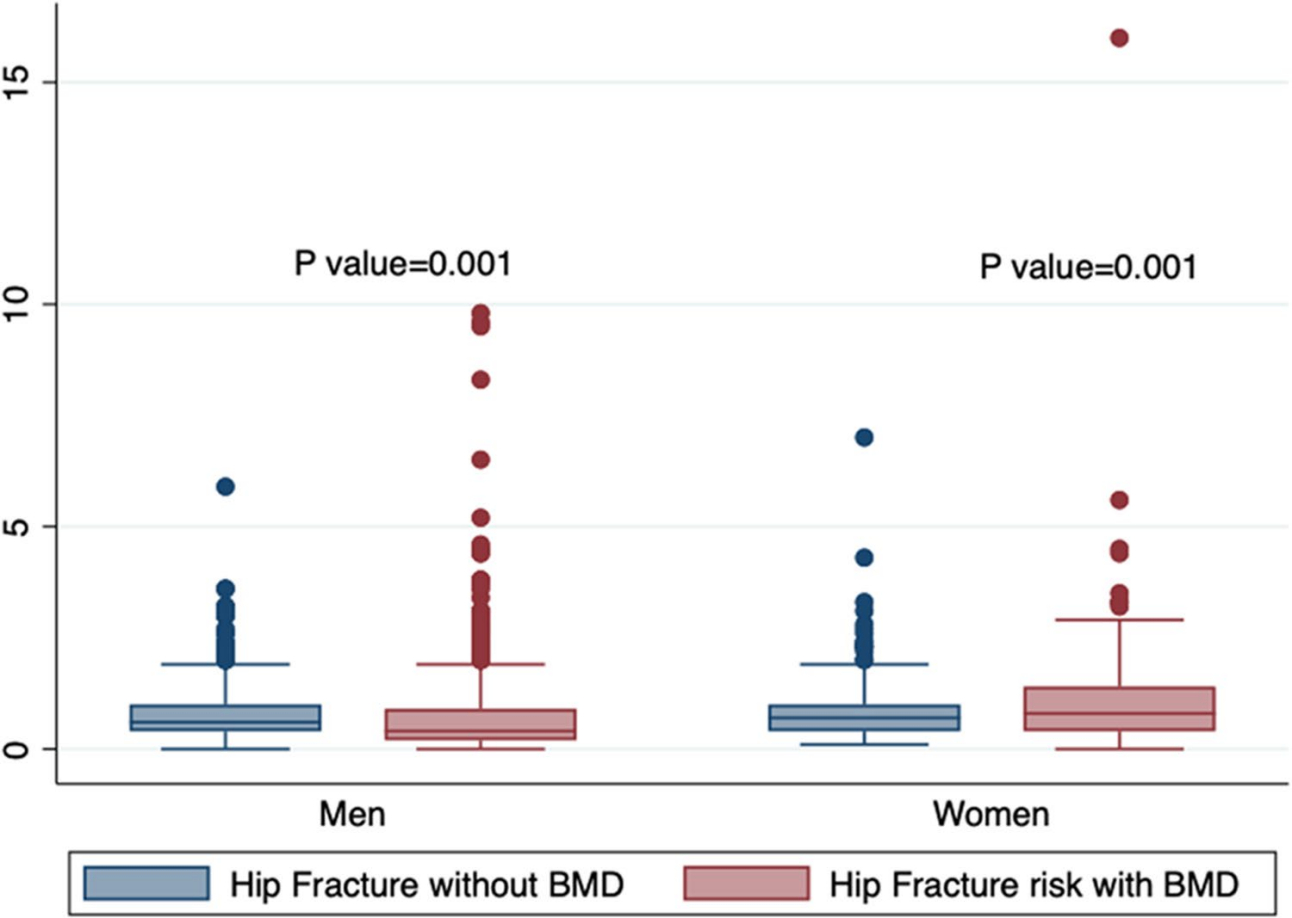
Comparison of hip fracture risk with and without bone mineral density by sex

This boxplot in Fig. 1 shows that including bone mineral density (BMD) in hip fracture risk prediction significantly increases the estimated risk for both men and women (*p* = 0.001). Median risks are higher with BMD included.

**Fig. 2:**
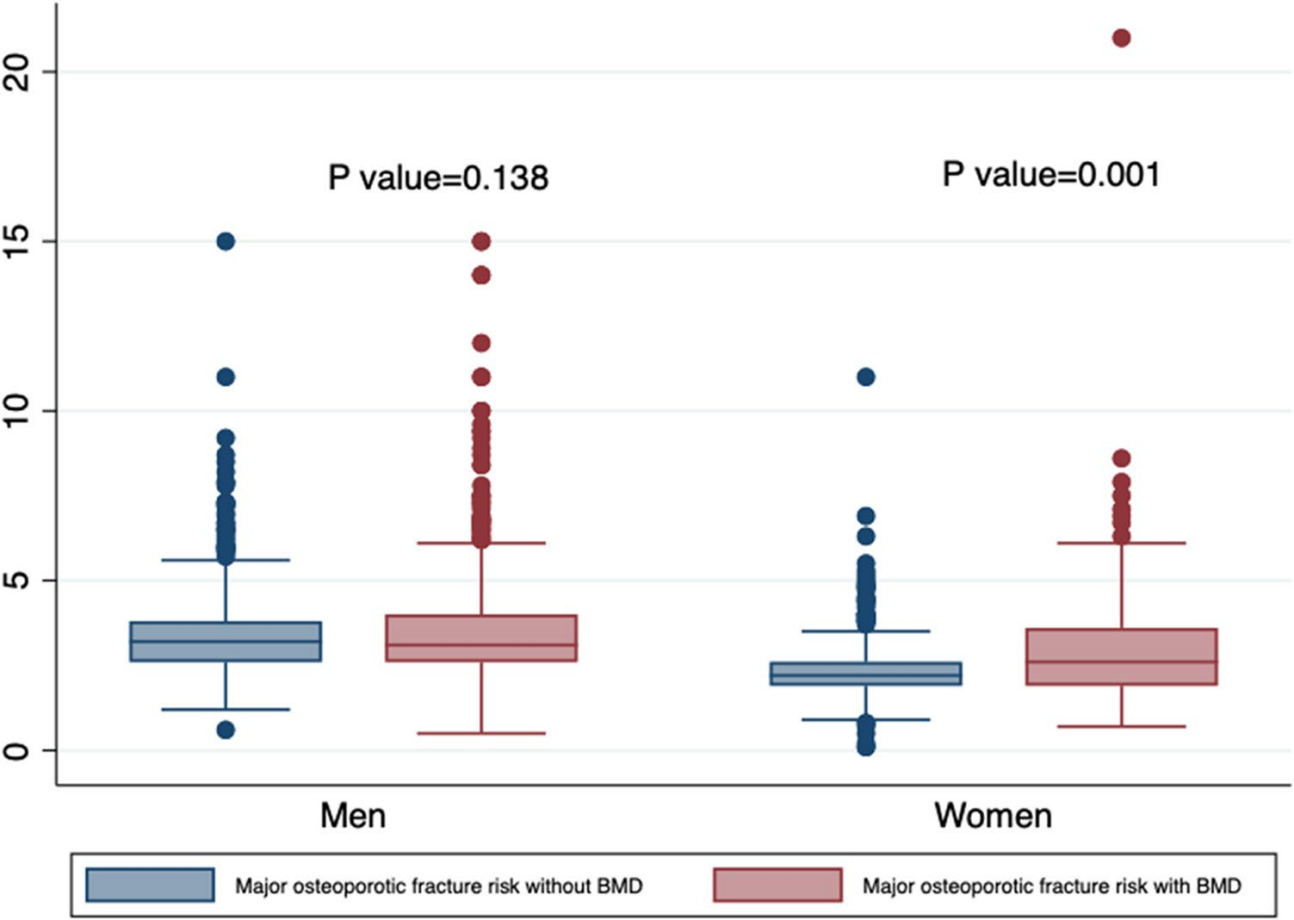
Comparison of major osteoporotic fracture risk with and without bone mineral density by sex

The boxplot in Fig. 2 compares major osteoporotic fracture risk with and without including bone mineral density (BMD) for men and women. While the inclusion of BMD significantly increases risk estimates for women (*p* = 0.001), the difference is not statistically significant for men (*p* = 0.138). Median risks are generally higher with BMD.

Incorporating BMD into FRAX calculations resulted in only modest shifts in risk classification (Table 4). For HF risk, 1.2% of participants were reclassified: 17 (1.19%) moved from low risk (≤ 3%) to high risk (> 3%), while an equal number shifted from high to low risk. For MOF risk, reclassification occurred in 0.49% of cases, with seven participants moving from low risk (< 10%) to higher categories—six into the moderate (10%–20%) group and one into the high (> 20%) group. Overall, these results suggest that incorporating BMD into FRAX has a limited impact on risk stratification, with only a small subset of individuals shifting into higher-risk categories.

**Table 4:** Comparison of FRAX score categories for HF and MOF risk with and without BMD, including reclassification rate (%) and count (n)

FRAX score of HF category	Without BMD (n = 1429)	With BMD (n = 1429)	Reclassification rate (%)
Low risk (≤ 3%)	1,417 (99.16%)	1,400 (97.97%)	** − 17 (− 1.20%)**
High risk (> 3%)	12 (0.84%)	29 (2.03%)	** + 17 (+ 1.19%)**
FRAX score of MOF category			
Low risk (< 10%)	1,426 (99.79%)	1,419 (99.30%)	− 7 (− 0.49%)
Moderate risk (10%–20%)	3 (0.21%)	9 (0.63%)	+ 6 (+ 0.42%)
High risk (> 20%)	0 (0.00%)	1 (0.07%)	+ 1 (+ 0.07%)

Age-specific comparisons of FRAX-based risk classification for MOF and HF using guideline-defined clinical categories are presented in Tables 5 and 6. For MOF risk (Table 5), incorporating BMD produced a modest upward shift in risk distribution across all age groups. The proportion classified as low risk (no BMD required) declined from 63.1% to 57.9%, while the intermediate-risk category (further BMD assessment recommended) increased from 36.2% to 40.5%. The high-risk group (treatment indicated regardless of BMD) more than doubled, rising from 0.7% (10 individuals) to 1.6% (23 individuals). The most notable absolute reclassification occurred in the 60–64 age group, where 12 participants moved into the high-risk category after BMD was incorporated.

**Table 5:** Risk category counts and percentages by age group (based on FRAX with and without BMD for MOF)

Age (years)	n	FRAX scores of MOF without BMD (n = 1429)			FRAX scores of MOF with BMD (n = 1429)		
		Low risk (No BMD needed)	Intermediate risk(needs BMD)	High risk (treat without BMD)	Low risk (no BMD needed)	Intermediate risk (needs BMD)	High risk (treat without BMD)
60–64	536	269 (18.82%)	262 (18.34%)	5 (0.35%)	221 (15.47%)	303 (21.20%)	12 (0.84%)
65–69	380	250 (17.50%)	128 (8.96%)	2 (0.14%)	227 (15.88%)	148 (10.36%)	5 (0.35%)
70–74	249	177 (12.39%)	71 (4.97%)	1 (0.07%)	170 (11.89%)	76 (5.32%)	3 (0.21%)
75–79	166	130 (9.10%)	35 (2.45%)	1 (0.07%)	128 (8.96%)	36 (2.52%)	2 (0.14%)
80–84	69	51 (3.57%)	17 (1.19%)	1 (0.07%)	54 (3.78%)	14 (0.98%)	1 (0.07%)
85–89	29	25 (1.75%)	4 (0.28%)	0 (0.00%)	27 (1.89%)	2 (0.14%)	0 (0.00%)
Total	1429	902 (63.12%)	517 (36.19%)	10 (0.70%)	827 (57.90%)	579 (40.53%)	23 (1.61%)

**Table 6:** Risk category counts and percentages by age group (based on FRAX without BMD for HF)

Age (years)	n	FRAX scores of HF without BMD (n = 1429)			FRAX scores of HF with BMD (n = 1429)		
		Low risk (no BMD needed)	Intermediate risk (needs BMD)	High Risk (treat without BMD)	Low risk (no BMD needed)	Intermediate risk (needs BMD)	High risk (treat without BMD)
60–64	536	201 (14.07%)	323 (22.61%)	12 (0.84%)	281 (19.67%)	208 (14.56%)	47 (3.29%)
65–69	380	211 (14.77%)	157 (10.99%)	12 (0.84%)	231 (16.17%)	123 (8.61%)	26 (1.82%)
70–74	249	135 (9.45%)	110 (7.70%)	4 (0.28%)	151 (10.57%)	89 (6.23%)	9 (0.63%)
75–79	166	105 (7.35%)	57 (3.99%)	4 (0.28%)	111 (7.77%)	46 (3.22%)	9 (0.63%)
80–84	69	44 (3.08%)	21 (1.47%)	4 (0.28%)	50 (3.50%)	15 (1.05%)	4 (0.28%)
85–89	29	24 (1.68%)	4 (0.28%)	1 (0.07%)	27 (1.89%)	2 (0.14%)	0 (0.00%)
Total	1429	720 (50.39%)	672 (47.03%)	37 (2.59%)	851 (59.56%)	483 (33.80%)	95 (6.65%)

For HF risk (Table 6), the inclusion of BMD similarly shifted classifications toward higher-risk categories. The proportion of individuals identified as high risk increased from 2.6% (37 participants) to 6.7% (95 participants), while the intermediate-risk category declined from 47.0% to 33.8%. This trend was consistent across all age groups, with the most substantial change observed in the 60–64 age group, where the number of individuals classified as high risk rose sharply from 12 to 47.

## Discussion

Our results indicate that adding BMD to FRAX calculations influences fracture risk classification, especially for HFs and among younger age groups. Although the changes in median FRAX scores were relatively small, their statistical significance was consistent. Significant differences were observed between FRAX scores with and without BMD for both major osteoporotic and HFs; however, the absolute score differences remained minor, and the rate of clinical reclassification was low.

For HF risk, adding BMD increased the proportion of individuals classified as high risk from 0.84% to 2.03%. While this difference was statistically significant (*p* = 0.0005), it involved only 17 individuals (1.2%), indicating minimal practical impact at the population level. Likewise, for MOF risk, only 0.49% of participants were reclassified to higher-risk categories, with just one individual shifting into the high-risk group. This modest increase is likely explained by the study population, composed of older adults attending family medicine clinics, who already represent a group enriched with osteoporosis risk factors such as postmenopausal status and chronic comorbidities.

Age-stratified analyses add further perspective to these results. While statistically significant differences emerged in certain groups (≥ 60, 65, and > 80 years), the extent of individual reclassification remained minimal, particularly for MOF. Even among older adults, who are generally more susceptible to bone density–related risk shifts, incorporating BMD did not consistently alter classifications across treatment thresholds.

These findings indicate that FRAX without BMD is a useful and practical first step for risk stratification, particularly in settings with limited access to DXA. Nonetheless, incorporating BMD may add value in select cases—especially when clinical risk estimates lie near treatment thresholds—by refining risk assessment and guiding more tailored management decisions. For most patients, however, particularly those clearly classified as low or high risk based on clinical factors alone, the added impact of BMD on risk categorization appears modest.

These findings are consistent with the SOS guidelines, which advocate a stepwise strategy for fracture risk assessment using the Saudi-specific FRAX tool. According to these recommendations, initial risk estimation should be conducted without BMD, with BMD reserved for patients in the intermediate-risk category to guide treatment decisions. Individuals classified as low risk do not require additional testing, while those at high risk may initiate treatment without further DXA evaluation. This structured approach promotes efficient resource allocation and ensures that DXA use is concentrated on patients who are most likely to benefit from refined risk stratification [19].

Our findings reinforce this approach, indicating that although BMD may refine risk estimates in certain cases, its overall influence on reclassification and treatment decisions is limited for the majority of patients. FRAX without BMD remains a practical and reliable tool for population-level risk assessment, particularly in healthcare settings where access to DXA scanning is limited.

These findings align with several previously published studies. Simpkins et al. reported that FRAX estimates without BMD produced identical treatment recommendations as FRAX with BMD in 82.4% of male veteran patients. The mean age of patients with concordant recommendations was 67.9 ± 10.2 years, compared with 62.2 ± 8.9 years among those with differing recommendations (*p* = 0.011) [20]. Similarly, Gadam et al. found that in 151 subjects, 127 (84%) had identical fracture risk predictions regardless of whether BMD was included in the FRAX calculation. Of these, 30 met treatment criteria and 97 did not, with risk categorization unchanged between the two approaches. Age was the only factor significantly different between patients with concordant versus discordant predictions (median ages 64.42 and 76.25 years, respectively; *p* < 0.001) [21]. Kim et al. observed that in men, FRAX probabilities without BMD were lower than those with BMD for ages 50–59, similar for ages 60–69, and higher for ages 70 and above. In women, FRAX scores without BMD were generally lower than those calculated with BMD [22]. Likewise, Asirvatham et al. reported that FRAX without BMD yielded the same risk prediction as FRAX with BMD in 86.61% of cases. They also found that FRAX without BMD performed better in predicting fracture risk among younger patients with higher BMI, greater BMD, and a prior fracture history [23]. Collectively, these studies are consistent with our results, highlighting age as a key factor influencing FRAX outcomes when BMD is considered, particularly for estimating MOF risk in younger age groups.

This is also consistent with the U.S. Preventive Services Task Force recommendation to begin DXA screening at age 65 in women, reinforcing the view that routine BMD testing may not be necessary in younger, low-risk populations [24].

In summary, FRAX provides a reliable tool for estimating osteoporotic fracture risk, regardless of whether BMD is included. Multiple studies demonstrate that BMD rarely alters risk classification, particularly among younger individuals and those with higher BMI [21–23]. This supports the use of FRAX without BMD as an appropriate first step in risk assessment.

In settings with limited access to DXA, FRAX without BMD serves as a practical and cost-effective tool for guiding clinical decision-making [15, 23]. Even in well-resourced healthcare systems, BMD testing may be best reserved for patients whose risk estimates fall near treatment thresholds rather than applied universally. This strategy enhances efficiency, preserves clinical accuracy, and promotes more targeted allocation of healthcare resources (15).

A key strength of our study is its large sample size, which enhances the generalizability of the findings to the wider population. The use of multiple analytical approaches also allowed for a more comprehensive evaluation of differences between FRAX assessments with and without BMD. Nonetheless, several limitations should be acknowledged. The retrospective design introduces the potential for bias. Moreover, the study was limited to comparing FRAX estimates with and without BMD, without evaluating the overall predictive accuracy or performance of the FRAX tool itself. Finally, other factors that may influence FRAX predictions were not included in the analysis.

## Conclusion

FRAX is a practical tool for estimating osteoporotic fracture risk, with or without incorporating BMD. While the addition of BMD produced some differences in FRAX scores, it resulted in reclassification for only a small proportion of individuals—most notably in the 60–75 age group for HFs and the 70–75 age group for MOFs. This limited effect reinforces the role of FRAX without BMD as a reliable initial step in fracture risk assessment. It is also consistent with current guidelines, which recommend starting with clinical risk factors and reserving BMD testing for cases near decision thresholds. Such an approach ensures efficient resource use while maintaining accuracy and individualized risk stratification.

## Data Availability

The data are not publicly available due to patient privacy regulations but can be provided by the corresponding author upon reasonable request and institutional approval.
